# Salivary SARS-CoV-2 load reduction with mouthwash use: A randomized pilot clinical trial

**DOI:** 10.1016/j.heliyon.2021.e07346

**Published:** 2021-06-18

**Authors:** Fernanda de Paula Eduardo, Luciana Corrêa, Debora Heller, Carlo Amorin Daep, Carlos Benitez, Zilson Malheiros, Bernal Stewart, Maria Ryan, Clarisse Martins Machado, Nelson Hamerschlak, João Renato Rebello Pinho, Letícia Mello Bezinelli

**Affiliations:** a Hospital Israelita Albert Einstein, São Paulo, Brazil; b School of Dentistry, University of São Paulo, São Paulo, Brazil; c Postgraduate Program in Dentistry, Universidade Cruzeiro do Sul, São Paulo, Brazil; d Department of Periodontology, School of Dentistry, The University of Texas Health Science Center at San Antonio, San Antonio, USA; e Colgate-Palmolive Technology Center, Piscataway, NJ, USA; f Latin American Oral Health Association, São Paulo, Brazil; g Virology Laboratory, Institute of Tropical Medicine, University of São Paulo School of Medicine, Brazil

**Keywords:** SARS-CoV-2, Saliva, Antimicrobial agents, Hydrogen peroxide, Chlorhexidine gluconate, Cetylpyridinium

## Abstract

The saliva of patients with COVID-19 has a high SARS-CoV-2 viral load. The risk of spreading the virus is high, and procedures for viral load reduction in the oral cavity are important. Little research to date has been performed on the effect of mouthwashes on the salivary SARS-CoV-2 viral load. This pilot randomized single-center clinical trial investigated whether three types of mouthwash with solutions containing either 0.075% cetylpyridinium chloride plus 0.28% zinc lactate (CPC + Zn), 1.5% hydrogen peroxide (HP), or 0.12% chlorhexidine gluconate (CHX) reduce the SARS-CoV-2 viral load in saliva at different time points. Sixty SARS-CoV-2-positive patients were recruited and randomly partitioned into a placebo (oral rinsing with distilled water) group and other groups according to the type of mouthwash. Saliva samples were collected from the participants before rinsing (T0), immediately after rinsing (T1), 30 min after rinsing (T2), and 60 min after rinsing (T3). The salivary SARS-CoV-2 viral load was measured by qRT-PCR assays. Rinsing with HP and CPC + Zn resulted in better reductions in viral load, with 15.8 ± 0.08- and 20.4 ± 3.7-fold reductions at T1, respectively. Although the CPC + Zn group maintained a 2.6 ± 0.1-fold reduction at T3, this trend was not observed for HP. HP mouthwash resulted in a significant reduction in the SARS-CoV-2 viral load up to 30 min after rinsing (6.5 ± 3.4). The CHX mouthwash significantly reduced the viral load at T1, T2, and T3 (2.1 ± 1.5-, 6.2 ± 3.8-, and 4.2 ± 2.4-fold reductions, respectively). In conclusion, mouthwash with CPC + Zinc and CHX resulted in significant reductions of the SARS-CoV-2 viral load in saliva up to 60 mins after rinsing, while HP mouthwash resulted in a significant reduction up to 30 mins after rinsing. Despite this transitory effect, these results encourage further studies and suggest that these products could be considered as risk-mitigation strategies for patients infected with SARS-CoV-2.

## 1 Introduction

The World Health Organization classifies the severe acute respiratory syndrome coronavirus 2 (SARS-CoV-2), which causes coronavirus disease 2019 (COVID-19), as an airborne pathogen transmitted between individuals upon exposure to infected droplets and aerosols or contact with fomites [1]. In the absence of effective treatments, most countries have focused on measures of limiting viral transmission to control the spread of the disease. These practices include social distancing, mask-wearing, hand washing, quarantining, and contact tracing, although these protective measures have varying success rates [2]. While these precautions may be appropriate for the general public, SARS-CoV-2 routes of transmission place frontline medical providers and dental professionals at a higher risk of infection [3]. Health care providers are often in close proximity to their patients and will need to remove a patient's mask in order to conduct oral examination and dental procedures known to generate aerosol [4, 5, 6, 7, 8].

Current studies suggest the role of the oral cavity as a reservoir for SARS-CoV-2, with a number of reports showing elevated salivary viral titers among COVID-19-positive individuals [9, 10, 11, 12]. Other investigations have demonstrated the presence of SARS-CoV-2 in the gingival crevicular fluid [13] and in cells of the oral mucosa and salivary glands [14]. These findings suggest that the mouth represents an important route of SARS-CoV-2 transmission and that it should be targeted as part of a strategy to reduce the risk of transmission and to mitigate the spread of SARS-CoV-2.

Mouthwash containing chlorhexidine (CHX), hydrogen peroxide (HP), and cetylpyridinium chloride and zinc lactate (CPC + Zn) are often used in oral care and have been shown to reduce the number of bacteria in the oral cavity [15, 16] and are routinely used in the dental office as pre-procedural rinses [17, 18]. While the use of these active agents against viruses is much less studied, recent *in vitro* and *in silico* findings have suggested that these types of mouthwash could have clinical anti-SARS-CoV-2 effects [19, 20, 21, 22]. Previous epidemics of respiratory viral infections similar to COVID-19, which include SARS, MERS, and H5N1, have shown that using antiseptic mouthwash/solutions containing chlorhexidine gluconate (CHX) or polyvinylpyrrolidone iodine (PVP-I) can reduce the viral load in the mouth [23]. These active ingredients have been used in dental and healthcare settings extensively; therefore, they serve as evidence of the perceived value of rinsing as a precautionary step to reduce oral viral loads. HP solutions are strongly recommended for surface disinfection due to their property of disruption of the SARS-CoV-2 lipidic envelope, promoting the eradication of the virus on surfaces [24]; however, a clinical study did not reveal any positive effect of a 1% HP solution in SARS-CoV-2 viral load reduction in saliva [25]. CHX had a transient effect on the SARS-CoV-2 viral load reduction in saliva; however, this study was conducted on a small number of patients [26]. Although CPC is a quaternary ammonium compound with a promising effect against SARS-CoV-2 [27], no clinical study has been conducted yet on the action of this solution in the oral cavity. An exploratory clinical study [28] investigated the different types of commercial mouthwash against SARS-CoV-2 with promising results; however, further investigation is warranted [29].

Due to the insufficient evidence of SARS-CoV-2 viral load reduction in saliva after the use of different types of mouthwash, this clinical study was conducted to determine if commercial products containing 1.5% HP, 0.12% CHX, and 0.075% CPC + 0.28% zinc can reduce the SARS-CoV-2 viral load in the saliva, as well as the time required for oral viral load recovery.

## 2 Materials and methods

This study was a randomized, double-blinded, placebo-controlled, single-center pilot clinical trial conducted according to the guidelines of the Declaration of Helsinki revised in 2013. The study protocol was approved by the National Ethics in Human Research Committee of Brazil (Protocol #37760820.0.0000.0071). Each patient signed an Informed Consent Form after being individually informed about the nature of the proposed treatment along with its risks and benefits. The methodology and results described here apply to patients hospitalized in negative-pressure rooms who used different types of mouthwash without mechanical ventilation (ClinicalTrial.gov, NCT04537962).

### 2.1 Participants and eligibility

Patients hospitalized in negative-pressure rooms at the Hospital Israelita Albert Einstein (HIAE), Brazil, between June 2020 and July 2020, were considered eligible for enrollment.

The inclusion criteria were:

• Age of 18–90 years
• Length of hospitalization up to 3 days
• Previously identified to be positive for SARS-CoV-2 as determined by nasal swabbing and qRT-PCR
• Adequate performance in the use of the different types of mouthwash
• Adequate performance of oral hygiene

Exclusion criteria included:

• No detection of SARS-CoV-2 by qRT-PCR at the time of recruitment
• Hospitalized in intensive care units
• Lesions in the oral mucosa
• Bleeding in the oral cavity that prevented the collection of samples
• History of allergy, irritations, or other side effects of the use of the test substances
• Use of the test substances or other oral antimicrobials 48 h before the baseline collection
• Non-adherence to the established protocol or inability to perform planned procedures

### 2.2 Trial design, allocation, and randomization

This is a pilot multi-arm parallel study designed to analyze the equivalence between the interventions. Participants were randomly allocated using a computer-generated randomization. Same number of participants was allocated per arm (12 per arm); however, participants with an undetectable viral load in saliva at baseline were excluded. A minimal sample size per arm was previously calculated and maintained.

### 2.3 Sample size estimation

Due to the limited information on conducting research with COVID-19 subjects at study initiation, an optimal power analysis to estimate optimal sample size was not feasible for this study. A preliminary estimate sample size [30] was performed considering that the research is a pilot study and adopting the following parameters: a two-fold reduction in the viral load as minimal detectable difference between T0 and the other time points; 80% upper confidence limit; 80% power; and 0.05 significant level. The total number of participants for each group is 7. Considering the risk of dropping out of the study, 12 participants were allocated per group.

### 2.4 Blinding

Codes were generated during the randomization and were concealed from all patients and laboratory staff directly involved in the study until statistical analyses were completed. The researchers responsible for monitoring the rinsing process and collecting saliva samples could not be blinded because the oral disinfection protocol was different for each oral solution.

### 2.5 Collection of clinical data

Study participants were recruited from the HIAE, Brazil, between June 2020 and July 2020. All clinical and laboratory assessments were conducted at the hospital. Data such as sex, age, presence of comorbidities, COVID-19 symptoms, oxygen saturation, and length of lung lesions detected by computerized tomography scans were collected from the patients’ medical records.

### 2.6 Interventions

The study involved the following interventions:

a) Placebo - distilled water
b) 0.075% Cetylpyridinium chloride, 0.28% Zinc lactate (CPC + Zn; Colgate Total 12®, Colgate-Palmolive Company, Brazil);
c) 1.5% HP (Peroxyl®, Colgate-Palmolive Company, USA)
d) 0.12% Chlorhexidine gluconate (CHX; PerioGard®, Colgate-Palmolive Company, Brazil)
e) 1.5% Hydrogen peroxide + 0.12% chlorhexidine (HP; Peroxyl® +CHX; PerioGard®, Colgate-Palmolive Company)

The patients were instructed by a senior dentist to rinse following manufacturers’ instructions and to spit out the solution after rinsing. The volumes and rinsing durations were as follows:

a) Placebo group: rinse with 20 mL for 1 min
b) CPC + Zn group: rinse with 20 mL for 30 s
c) HP group: rinse with 10 mL for 1 min
d) CHX group: rinse with 15 mL for 30 s
e) HP + CHX group: rinse with 10 mL of HP mouthwash for 1 min, followed by rinsing with 15 mL of CHX mouthwash for 30 s

### 2.7 Primary outcomes

This study measures the effects of the different types of mouthwash or a sequential usage of HP mouthwash followed by CHX mouthwash on reducing the viral loads in saliva. This was measured based on the cycle threshold (Ct) values and fold changes relative to the placebo and baseline.

### 2.8 Saliva collection, detection, and analyses of viral RNA

Unstimulated saliva was collected at baseline (T0), immediately after rinsing (T1), 30 min after rinsing (T2), and 60 min after rinsing (T3) using a sterile 50-mL tube [31]. All samples were immediately placed on ice and stored in a freezer (−80 °C) until RNA extraction.

### 2.9 RNA extraction and qRT-PCR analysis

Viral RNA was extracted from 200-μL saliva specimens using a QIAsymphony™ RNA Kit (QIAGEN, Hilden, Germany) following the manufacturer's instructions. Amplification of the SARS-CoV-2 N and ORF1ab genes was performed using a commercial COVID-19 qRT-PCR kit (XGen Master, Mobius Life Science Ltda, Pinhais, Brazil). Briefly, 2 μL of RNA template was added to 18 μL of amplification mixture and centrifuged (at 8000 rpm) for 20 s. Positive and negative controls from the manufacturer were also used. cDNA synthesis was performed using QuantStudio™ (ThermoFisher Scientific Inc, Massachusetts, USA) using the following reverse transcription conditions: 42 °C for 60 min, followed by 70 °C for 10 mins. qRT-PCR conditions were performed as follows: 95 °C for 15 s followed by 60 °C for 1 min for 45 cycles. Samples were classified as negative for SARS-CoV-2 when both N and ORF1ab primer-probe sets were detected >40 Ct. All the tests were performed in triplicate.

Double delta Ct (ΔΔCt) and fold change values were determined using Ct values collected from the amplification plots only for the N gene due to the variability in ORF1ab amplifications. The placebo group was considered as reference control (CTC), and baseline time (T0) was considered the experimental control (CTE). First, the difference between Ct values of placebo at T1, T2, and T3 in relation to T0 was determined, obtaining a reference delta (ΔCTC). Then, the difference between Ct values at T1, T2, and T3 in each mouthwash group in relation to T0 was calculated, obtaining an experimental delta (ΔCTE). ΔΔCt (fold-difference) was then determined using the formula *2*^*-(ΔCTC-ΔCTE)*^. Fold reduction was calculated using *1/ΔΔCt*. A fold change cut-off value ≥ 2 was considered a significant reduction [32, 33]. Patients with no detectable virus in their saliva after rinsing with the mouthwash were not included in the calculation of fold differences.

### 2.10 Collection of clinical data

Study participants were recruited from the HIAE, Brazil, between June 2020 and July 2020. All clinical and laboratory assessments were conducted at the hospital. Data such as sex, age, presence of comorbidities, COVID-19 symptoms, oxygen saturation, and length of lung lesions detected by computerized tomography scans were collected from the patients’ medical records.

### 2.11 Statistical analysis

Due to the limited information on conducting research with COVID-19 subjects at study initiation, power analysis to estimate the optimal sample size was not feasible for this study. For clinical information, categorical data are presented as absolute and relative (%) frequencies, and the numerical data are presented as the median, minimum, and maximum values. The statistical analysis for Ct values was exploratory. Differences in the Ct values of T1, T2, and T3 were compared with T0 in each group separately using generalized estimating equations. Bonferonni-adjusted 95% confidence intervals were performed on the Ct values to control the simultaneous confidence level for an entire set of confidence intervals, maintaining simultaneous confidence levels to counter the higher error rate. The confidence level for each individual interval was adjusted so that the resulting simultaneous confidence level is equal to the specified value.

## 3 Results

### 3.1 Participants' flow and recruitment

**Figure 1:**
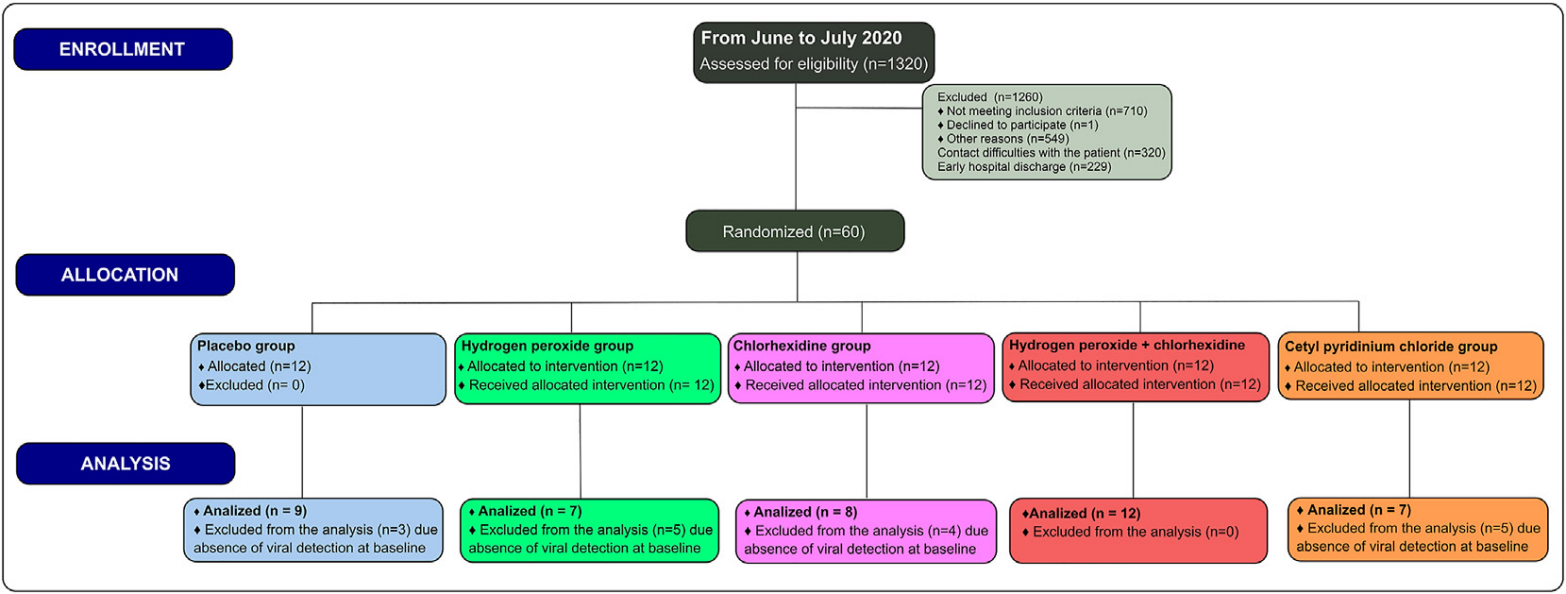
Flow diagram of the patients.

Sixty patients were recruited at random for this study (Figure 1), and twelve patients were assigned to each group. Subjects with undetectable viral loads in saliva at baseline (T0) were excluded from the analysis. As a result of this adjustment, 9 patients remained in the placebo group, 7 patients each in the HP and CPC + Zn groups, 8 patients in the CHX group, and all twelve patients remained for the group who sequentially rinsed with HP followed by CHX.

### 3.2 Baseline data

The patients’ recorded clinical data are provided in Table 1. In all groups, the majority of patients were men; the median age ranged from 46 years to 62 years. A majority of the participants (75%) had at least a risk factor for COVID-19 complications. All the patients exhibited mild-to-moderate COVID-19 symptoms, fatigue, cough, nasal congestion, and dyspnea being the predominant symptoms. The median day of symptom onset varied from 2 to 7 days. Although two patients had oxygen saturation levels of < 90%, no patient in this study required any mechanical ventilation. It was also noted that all the patients exhibited lung lesions, with extension up to 50%, but maintained the non-severe form of the disease during the study [34].

**Table 1:** Clinical characteristics of the patients during sample collection.

	Groups					P value
	Placebo (n = 9)	CPC + Zn (n = 7)	HP (n = 7)	CHX (n = 8)	HP + CHX (n = 12)	
**Male**–n (%)	6 (66.7)	5 (71.4)	4 (57.1)	7 (87.5)	10 (83.3)	0.620
**Female**–n (%)	3 (33.3)	2 (28.6)	3 (42.9)	1 (12.5)	2 (16.7)	
**Age** - median (range)	59 (36–85)	46 (34–88)	62 (40–87)	53.5 (49–88)	53 (40–72)	0.564
**Number of risk comorbidities** - median (range)	1 (0–4)	2 (0–3)	1 (0–5)	1 (0–4)	1 (0–3)	0.629
Hypertension–n (%)	2 (22.2)	3 (42.9)	3 (42.9)	2 (25.0)	3 (25.0)	
Heart/circulatory diseases–n (%)	3 (33.3)	1 (14.3)	1 (14.3)	2 (25.0)	1 (8.3)	
Diabetes–n (%)	2 (22.2)	3 (42.9)	3 (42.9)	1 (12.5)	1 (8.3)	
Lung/respiratory diseases–n (%)	1 (11.1)	0 (0.0)	1 (14.3)	1 (12.5)	2 (16.7)	
Renal disease–n (%)	0 (0.0)	2 (28.6)	1 (14.3)	2 (25.0)	0 (0.0)	
Obesity–n (%)	2 (22.2)	2 (28.6)	0 (0.0)	0 (0.0)	2 (16.7)	
Hypothyroidism–n (%)	1 (11.1)	0 (0.0)	1 (14.3)	0 (0.0)	2 (16.7)	
**COVID-19 related symptoms–**n (%)						
Fatigue	9 (100.0)	6 (85.7)	7 (100.0)	7 (87.5)	11 (91.7)	
Fever >37.5 °C	6 (66.7)	7 (100.0)	4 (57.1)	5 (62.5)	7 (58.3)	
Headache	3 (33.3)	4 (57.1)	2 (28.6)	2 (25.0)	6 (50.0)	
Cough	7 (77.8)	5 (71.4)	4 (57.1)	5 (62.5)	12 (100.0)	
Diarrhea	2 (22.2)	3 (42.9)	2 (28.6)	2 (25.0)	1 (8.3)	
Nasal congestion	3 (33.3)	7 (100.0)	7 (100.0)	6 (75.0)	10 (83.3)	
Taste changes	1 (11.1)	3 (42.9)	3 (42.9)	1 (12.5)	5 (41.7)	
Xerostomia	7 (77.8)	3 (42.9)	4 (57.1)	4 (50.0)	5 (41.7)	
Dyspnea	3 (33.3)	7 (100.0)	4 (57.1)	6 (75.0)	10 (83.3)	
Days of symptoms onset–median (range)	4 (2–10)	2 (1–6)	7 (2–11)	4.5 (3–10)	5.5 (2–15)	0.274
**O**_**2**_**saturation (%)**–median (range)	95 (92–99)	96 (88–99)	94 (91–97)	96 (92–99)	92 (90–96)	0.563
**Extension of lung lesion (%)–**n (%)						
<25%	4 (44.4)	2 (28.6)	2 (28.6)	5 (62.5)	4 (33.3)	0.600
25%–50%	5 (55.6)	5 (71.4)	5 (71.4)	3 (37.5)	8 (66.7)	

HP = hydrogen peroxide; CHX = chlorhexidine gluconate; CPC + Zn = cetylpyridinium chloride, Zinc lactate.

P-values calculated using the χ2 test for categorical data and the ANOVA test for continuous data.

### 3.3 Outcomes and estimation

**Figure 2:**
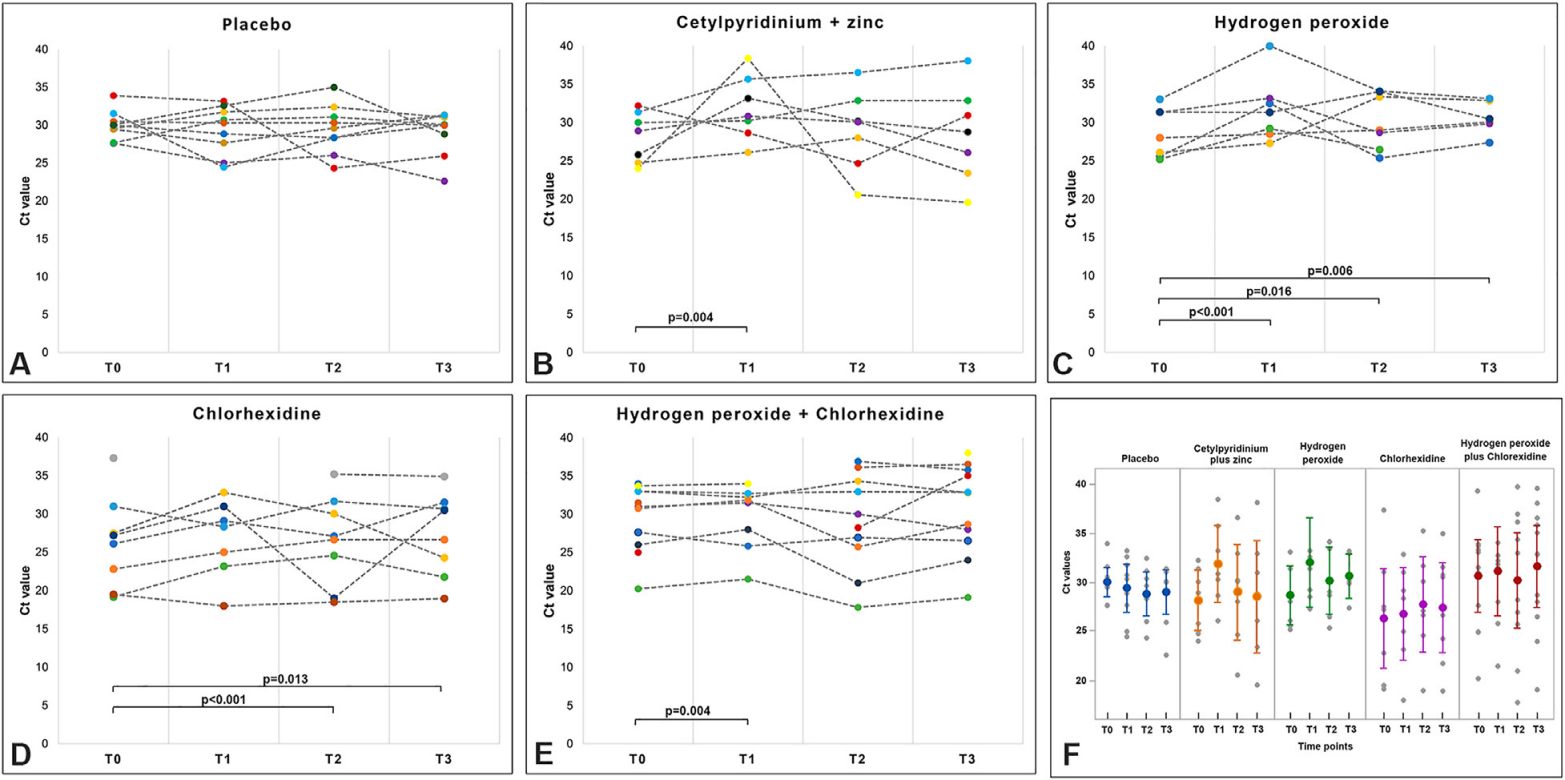
A to E: Cycle Threshold (Ct) values in the saliva of COVID-19-positive patients treated with mouth rinses in accordance with the baseline (T0), immediately after the rinsing (T1), 30 min (T2), and 60 min (T3) after the rinsing. The color dots represent Ct values for each patient. The interrupted line represents the undetected viral load. P-values obtained using generalized estimating equations with Bonferroni test adjustments (5% significance level). F: The grey dots represent Ct values for each patient. The color dot represents the mean value, and the vertical bar represents the Bonferonni-adjusted 95% confidence intervals.

Based on the threshold used for the qRT-PCR analysis, the Ct values for each treatment are shown in Figure 2. Whenever the mean Ct value for each experimental group was compared to the baseline value, a significant difference was observed for CPC + Zn at T1 (p = 0.004); in the HP group at T1 (p < 0.001), T2 (p = 0.016), and T3 (p = 0.006); in the CHX group at T2 (p < 0.001) and T3 (p = 0.013); and in the HP + CHX group at T1 (p = 0.004). The HP and CPC + Zn mouthwashes exhibited higher Ct levels at T1 relative to T0, T2, and T3. The placebo mouthwash and HP + CHX mouthwash exhibited minor changes in Ct levels across T0, T1, T2, and T3. After the rinsing, the virus was undetectable in four patients at T1 (3 in the HP + CHX group and 1 in the CHX group), one at T2 (in the HP + CHX group), and one at T3 (in the HP group), suggesting an absence of the viral load at these time points.

**Figure 3:**
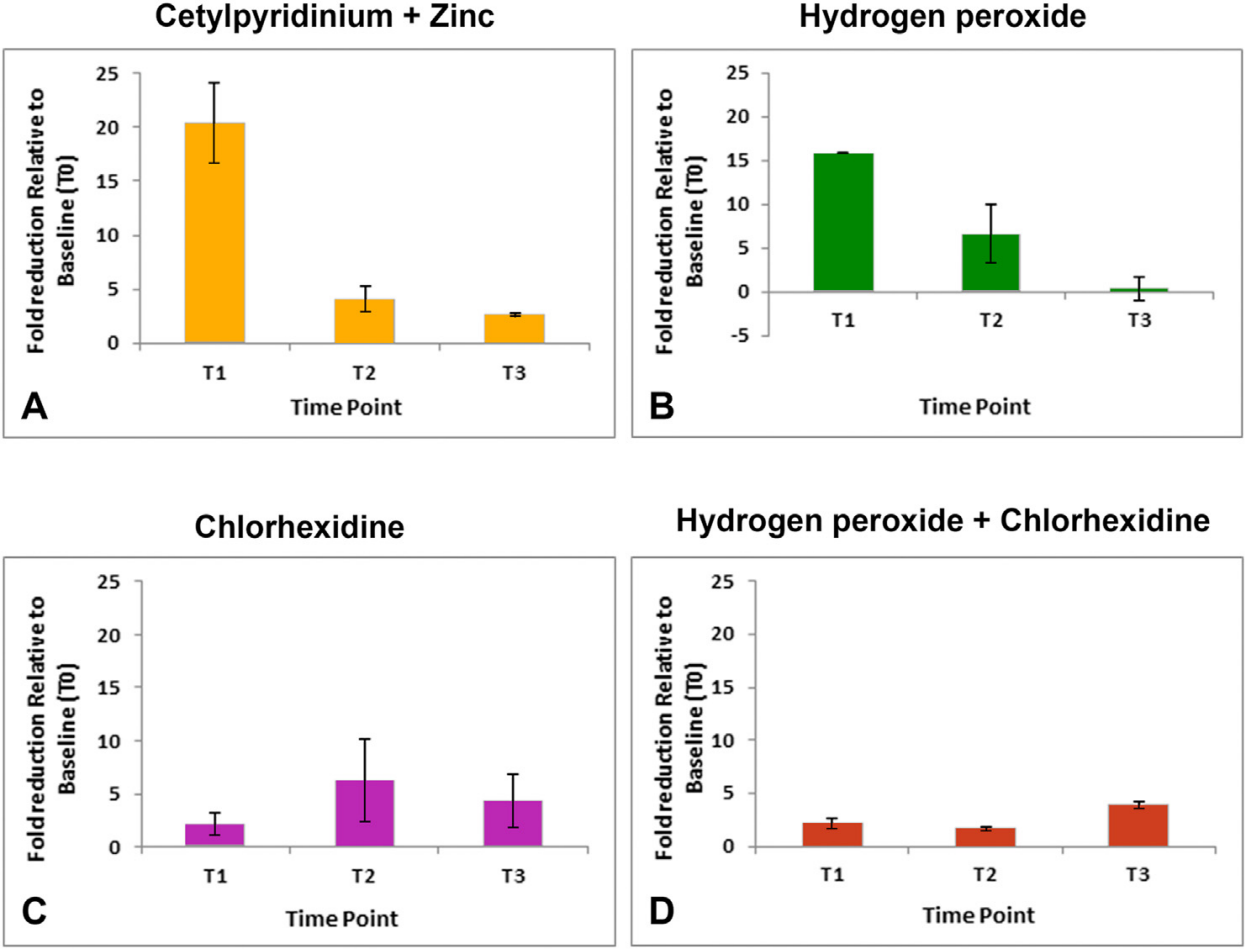
Mean (±standard deviation) of SARS-CoV-2 fold reduction in saliva immediately after rinsing (T1), 30 min after rinsing (T2), and 60 min after rinsing (T3). A reduction was considered significant when fold reduction was >2. Rinsing with the tested mouthwashes or combination of mouthwashes effectively reduced oral viral levels in COVID-19-positive patients at some time point. The highest reductions in viral loads were observed at T1 for the Cetylpyridinium plus Zinc mouthwash (A) and the Hydrogen Peroxide mouthwash (B); these rinses maintained levels of >2 viral load reduction at T2; the Chlorhexidine mouthwash (C) showed significant viral load reductions at all the time points, although the reductions were better at T2 and T3; although the combination of Hydrogen peroxide followed by Chlorhexidine (D) showed the lowest reductions, results >2 viral load reductions were achieved at T1 and T3.

The observed trends for Ct value changes were consistent with the calculated fold reductions relative to baseline values. Based on ΔΔCt values, all treatment groups were observed to have 2-fold or greater viral bioload reduction in saliva immediately after use (Figure 3).

Rinsing with the HP mouthwash or the CPC + Zn mouthwash showed the best reduction in viral load, with a 15.8 ± 0.08- and 20.4 ± 3.7-fold reduction immediately after rinsing at T0, respectively. Although the CPC + Zn group still maintained a 2.6 ± 0.1-fold less SARS-CoV-2 in saliva at T3, this trend was not observed for HP (0.3 ± 1.3-fold reduction). This mouthwash had a significant reduction up to 30 min after the rinsing (6.5 ± 3.4).

The CHX mouthwash results met the minimum acceptance criteria of a ≥ 2-fold reduction to show its effectiveness in viral load reduction at T1, T2, and T3 (2.1 ± 1.5 fold, 6.2 ± 3.8 fold, and 4.2 ± 2.4 fold reductions, respectively). The sequential use of the HP mouthwash followed by the CHX mouthwash did not lead to an increase in viral reduction efficacy at T2 (1.6 ± 0.2); 2.1 ± 0.5-fold and 3.9 ± 0.3-fold reductions were detected at T1 and T3, respectively.

### 3.4 Harms

None of the patients experienced discomfort or side effects after the interventions.

## 4 Discussion

This current clinical study showed that rinsing with three different types of mouthwash (CPC + Zn, HP, and CHX) temporarily reduced the bioload of SARS-CoV-2 in saliva among COVID-19-positive patients. These results are in line with the findings of Seneviratne *et al.* [28], which showed the role of different types of mouthwash in helping to reduce the salivary viral load.

The mechanism by which these products reduce the viral load in the oral cavity needs to be elucidated. The tested products may help reduce the viral load by destroying the viral envelope needed for binding to cells in the oral cavity and/or by mechanically flushing out the viruses found in saliva.

Like other viruses, such as influenza A and B, the SARS-CoV-2 envelope is similar in composition, consisting of lipids and glycosylated proteins that can be susceptible to disruption by cationic, amphiphilic compounds such as CPC [35]. The integration of these agents into the viral envelope will render it permeable, ultimately resulting in the neutralization of the virus. The current study demonstrated that the CPC + Zn mouthwash immediately reduced the SARS-CoV-2 viral load in the mouth. This study suggests the potential use of a CPC + Zn mouthwash as a risk-mitigation step to help reduce the oral viral load of SARS-CoV-2 among COVI1D-19-positive patients.

HP at a concentration of 1.5% is recognized as a debriding agent in dentistry that has antiseptic properties. The oxidative property of HP contributes to its antiseptic nature, specifically against viruses, by disrupting the viral envelope and degrading viral RNA [36]. The current study demonstrated that HP mouthwash was indeed effective in reducing the viral load in saliva immediately after rinsing, although the viral load returned to its baseline value within 60 mins post-rinsing. This finding is consistent with the known lack of substantivity of HP.

CHX-containing types of mouthwash have historically been used in the dental office to reduce infections associated with aerosolized bacteria [37]. A previous study demonstrated that a 0.12% CHX mouthwash was effective against viruses such as herpes, influenza, parainfluenza, and hepatitis B. This suggests that CHX-containing products may contribute significantly to mitigating oral levels of SAR-CoV-2 in the mouth [38], with the provided short-term reduction in SARS-CoV-2 titers tending to return to baseline within 2 h [39]. The results of the current study are consistent with their findings of significant reductions of the viral load occurring mainly after 30 min and 60 min. Additional clinical studies are required to better understand the effects of CHX against the virus and to further validate its effectiveness as a risk-mitigation strategy during the current pandemic.

COVID-19 patients who sequentially rinsed with HP mouthwash followed by CHX mouthwash did not obtain any additional benefits from this regimen, which resulted in a minimal observed reduction in the salivary viral load, even at T1. Secondary rinsing with CHX might have washed out the delivered HP in the mouth due to its reduced substantivity, reducing the contact time required for HP to deliver its antiviral effects. Better outcomes may be observed by switching the order of rinsing in future clinical studies by initially delivering CHX across the oral surfaces, followed by an HP rinse. It is interesting to note that some patients (4/11 patients, 36.3%) in the HP-CHX group had undetectable salivary SARS-CoV-2 levels after rinsing. While these patients could not be included in the fold change analysis, this could indicate the potential value for the sequential use of different types of mouthwash. Additional regimen studies are required to validate this clinically.

The removal of viruses found in the saliva of COVID-19-positive patients does not constitute a treatment for the disease, neither does it provide a permanent reduction in the oral viral load, as this study demonstrated. As SARS-CoV-2 replicates within the respiratory system, sputum from the upper respiratory tract harboring infectious viral particles can deliver the virus back into the oral cavity when a person talks or coughs. A limitation of this study was the absence of an analysis of the effect of the products on viruses bound to the surface of soft tissue and about the infectivity of any remaining treated viruses in the mouth after rinsing. The viral neutralization properties of each oral solution are poorly understood, and further in vitro and clinical studies are necessary. Laboratory assays for analysis of capsid disassembly and viral uncoating of SARS-CoV-2 exposed to these oral antimicrobial solutions may contribute to the knowledge about the mechanism of action of these products.

Other limitations were the small sample and the lack of information about patients’ oral conditions at baseline. Further clinical investigations with a larger study population would provide a better positive correlation on the benefits of oral rinsing in reducing the oral SARS-CoV-2 viral load. Future studies should consider collecting additional baseline data (such as a complete oral exam assessing periodontal status), including oral hygiene and plaque indices, along with a quantification of salivary flow. This information could contribute to a more comprehensive interpretation of the results.

Despite the relatively limited number of patients in the current study and the temporary effects of the products, the use of different types of mouthwash as an adjunct to current risk-mitigation practices of mask-wearing, handwashing, and social distancing, could be considered.

In conclusion, CPC + Zinc mouthwash and CHX mouthwash provided a significant reduction in the SARS-CoV-2 viral load in saliva up to 60 mins after rinsing, while HP provided a significant reduction up to 30 mins after rinsing. Despite this transitory effect, these results encourage further studies and suggest that these products could be considered as risk-mitigation strategies for patients infected with the SARS-CoV-2 virus.

## Declarations

### Author contribution statement

Fernanda de Paula Eduardo and Letícia Mello Bezinelli: Conceived and designed the experiments; Performed the experiments; Analyzed and interpreted the data; Contributed reagents, materials, analysis tools or data; Wrote the paper.

Luciana Corrêa: Conceived and designed the experiments; Analyzed and interpreted the data; Contributed reagents, materials, analysis tools or data; Wrote the paper.

Debora Heller and João Renato Rebello Pinho: Conceived and designed the experiments; Analyzed and interpreted the data; Wrote the paper.

Carlo Amorin Daep and Carlos Benitez: Analyzed and interpreted the data; Wrote the paper.

Zilson Malheiros, Bernal Stewart, Maria Ryan and Clarisse Martins Machado: Analyzed and interpreted the data; Contributed reagents, materials, analysis tools or data; Wrote the paper.

Nelson Hamerschlak: Conceived and designed the experiments; Wrote the paper.

### Funding statement

This work was supported by 10.13039/100004368Colgate-Palmolive Company.

### Data availability statement

Data will be made available on request.

### Declaration of interests statement

The authors declare the following conflict of interests: Zilson Malheiros, Bernal Stewart, Carlo Amorin Daep and Maria Ryan are currently employed by Colgate-Palmolive Company.

### Additional information

The clinical trial described in this paper was registered at ClinicalTrials.gov under the registration number NCT04537962.
